# A call to action: improving access to cardiac MRI for diagnosis of immune checkpoint inhibitor related myocarditis in low and middle income countries

**DOI:** 10.1186/s40959-025-00393-8

**Published:** 2025-10-30

**Authors:** Aruni Ghose, Abhinav Kandala, Isha Sabnis, Maryam Hasanova, Carl Simela, Charlotte Manisty, Suvro Banerjee, Sebastian Szmit, Mark Westwood, Ariane V. S. Macedo, Daniel Sierra-Lara Martinez, Andrès J. Daniele, Vanita Noronha, Trishun Singh, Bonnie Ky, Sola Adeleke, Akash Maniam, Stephen J. Casselli, Daniel J. Lenihan, Susan Dent, Arjun Kumar Ghosh

**Affiliations:** 1 Cardio-Oncology Service, Barts Heart Centre, St Bartholomew’s Hospital, Barts Health NHS Trust, London, UK; 2 Barts Cancer Centre, St Bartholomew’s Hospital, Barts Health NHS Trust, London, UK; 3 Immuno-Oncology Clinical Network, Liverpool, UK; 4 United Kingdom and Ireland Global Cancer Network, Manchester, UK; 5 International Cardio-Oncology Society, Tampa, FL USA; 6 Asian Cardio-Oncology Society, Mumbai, India; 7 University College London Medical School, London, UK; 8 Hatter Cardiovascular Institute, University College London, London, UK; 9 School of Medicine and Population Health, University of Sheffield, Sheffield, UK; 10 Division of Biosciences, University College London, London, UK; 11 Cardio-Oncology Service, University College London Hospitals NHS Foundation Trust, London, UK; 12 Institute of Cardiovascular Sciences, University College London, London, UK; 13 Department of Cardiology, Apollo Multispeciality Hospitals, Kolkata, India; 14 Department of Cardio-Oncology, Centre of Postgraduate Medical Education, Institute of Hematology and Transfusion Medicine, Warsaw, Poland; 15 Clinic of Oncological Diagnostics and Cardio-Oncology, Maria Skłodowska-Curie National Research Institute of Oncology, Warsaw, Poland; 16 Cardio-Oncology Clinic, Santa Casa de São Paulo School of Medicine, São Paulo, Brazil; 17 Coronary Care Unit, Instituto Nacional de Cardiologia, Ciudad de Mexico, Mexico; 18 Cardio-Oncology Department, Instituto de Oncología “Ángel H. Roffo”, Buenos Aires, Argentina; 19 Asociación Cardio-Oncología de la República, Bulnes, Argentina; 20 Department of Medical Oncology, Tata Memorial Hospital, Tata Memorial Center, Homi Bhabha National Institute, Mumbai, India; 21 Cardio-Oncology Unit, Netcare Umhlanga Hospital, Durban, South Africa; 22 Cardio-Oncology Society of Southern Africa, Durban, South Africa; 23 Thalheimer Center for Cardio-Oncology, Abramson Cancer Center, Perelman School of Medicine, University of Pennsylvania, Philadelphia, USA; 24 School of Biomedical Engineering & Imaging Sciences, King’s College London, London, UK; 25 Caribbean Cancer Research Institute, El Socorro, Trinidad and Tobago; 26 Cardio-Oncology Program and Heart Failure Clinic, Cape Cardiology, Saint Francis Healthcare System, Cape Girardeau, MO USA; 27 Wilmot Cancer Institute, University of Rochester Medical Centre, Rochester, NY USA; 28 Cardio-Oncology Program, Department of Medicine, University of Rochester, Rochester, NY USA; 29 Canadian Cardiac Oncology Network, Toronto, ON Canada

## Abstract

Immune checkpoint inhibitor (ICI) therapy is a rapidly expanding pillar of cancer treatment, but it carries the risk of immune-related adverse events. Among the most fatal is ICI-related myocarditis (ICIRM). Cardiac magnetic resonance (CMR) imaging is the non-invasive current gold standard for diagnosis, with significant disparities regarding availability and utilisation. The vast majority of ICIRM cases are reported in high-income countries (HICs), reflecting not only patterns of ICI use, but also a profound diagnostic gap in low- and middle-income countries (LMICs). LMICs face barriers to CMR access, including a stark deficit of MRI scanners, with approximately 1 scanner per million people in LMICs versus 26 per million people in HICs, prohibitive costs, and a critical shortage of trained radiologists and cardiologists. The inequity means that as ICI therapy becomes increasingly accessible worldwide, patients in resource-limited settings will be at a high-risk of undiagnosed and untreated ICIRM. Our paper issues a call to address this critical healthcare disparity. To improve CMR access for ICIRM diagnosis in LMICs, a multi-pronged strategy is imperative – Governmental support and policy change to prioritise infrastructure investment and integrate CMR into national health strategies; targeted educational programmes, such as the SWiM and ‘train the trainer’ initiatives, to build local expertise in CMR acquisition and interpretation; adoption of technological innovations, including cost-effective rapid CMR protocols and artificial intelligence (AI) tools that can reduce scan times.

## Introduction to ICI related myocarditis

Immune Checkpoint Inhibitor (ICI) Related Myocarditis (ICIRM) is one of the least reported but most fatal immune related adverse events (IRAEs) of ICI therapy. ICIRM fatality rates can range from 20 to 50%. Their incidence can range from 1.4% on single agent to 2.4% on combination ICI [1, 2].

High income countries (HICs) like USA and those in Europe account for 54% and 33% of ICIRM reported cases respectively compared to Asian countries with 11% [1]. These reporting inequalities may result from barriers to ICIRM diagnosis including decreased exposure to ICI therapy and awareness of complications hence limiting cardiovascular evaluation, access to healthcare providers or even ICI treatment in lower-middle-income countries (LMICs). For example, a leading Indian (LMIC) tertiary cancer centre identified 15, 674 thoracic, head and neck cancer patients with a clinical indication for ICI therapy with only 3% receiving it [3]. Even in an upper-middle income country (UMIC) i.e., Armenia, only 23% of 185 ICI eligible metastatic lung cancer patients received ICI therapy, of which 34% discontinued treatment due to cost [4].

The expenditure on ICIs for cancer therapy has increased from $2.8 million to $4.1 billion between 2011 and 2021 respectively, indicating a surge in number of patients treated, given the ever-expanding Food and Drug Administration (FDA) indications [5]. ICIRM cases have also witnessed a surge from 3% in 2010 to 76% in 2017 [1]. Moreover, recent data show a 1-year mortality of 29% amongst severe vs. 5% amongst non-severe ICIRM presentations, potentially reflecting improvements in management strategies [6].

In summary, majority of ICIRM is currently reported in HICs. Even though differential rates of utilisation prevail, this is likely to change going forwards globally, and therefore variation in access to diagnostic tools will become increasingly relevant. In this regard, the diagnosis of ICIRM can be challenging and cardiac magnetic resonance imaging (CMR) plays a vital role in demonstrating myocardial inflammation or showing alternate diagnoses. While CMR is commonly used for this purpose in HICs, it is not as readily available in LMICs. We wish to issue a clarion call to improve the access to CMR in suspected cases of ICIRM in LMICs via the medium of this paper.

## Definition of ICIRM and the role of CMR in diagnosis

The 2022 International Cardio-Oncology Society (IC-OS) consensus statement defined ICIRM as “rise in cardiac troponin with presence of 1 major criterion or 2 minor criteria” after excluding differentials like acute coronary syndrome and infective myocarditis [7]. Cardiac MRI (CMR) is the established gold standard non-invasive diagnostic tool for ICIRM, although 50% of cases may have normal CMRs [8]. CMR “suggestive of acute myocarditis” finds itself as the only major criterion. It also features as 1/5 minor criteria, i.e., “suggestive CMR”. This implies usage of some of the modified Lake Louise criteria – T2-weighted image abnormalities indicating myocardial oedema; T1-weighted image abnormalities indicating myocardial injury i.e., increased myocardial T1 map value/increased extracellular volume/positive late gadolinium enhancement (LGE) [7].

Since the seminal case report of ICIRM published November 2016, 7 relevant clinical practice guidelines in cardio-oncology and IRAEs have been published – 6 from high income and 1 from upper middle income geographical regions [9]. These have been used as knowledge sources for mapping recommendations regarding status of CMR in ICIRM after comparative analysis using proprietary methods of OncoFlow™ (UK00004075205) (Table 1).

**Table 1:** This table uses the OncoFlow™ (UK00004075205) proprietary methods to map recommendations on the role of cardiac MRI in ICIRM on comparative analysis of clinical practice guidelines used as knowledge sources

Guideline [Reference number]	Organisation	Region of Origin (World Bank Income Group)	Year of Publication	Recommendation [Level of Evidence, Grade/Strength]
Management of cardiac disease in cancer patients throughout oncological treatment: ESMO consensus recommendations [13]	European Society for Medical Oncology (ESMO)	Europe (High Income)	2020	For patients who develop new CV symptoms or are incidentally noted to have any arrhythmia, conduction abnormality on ECG or LVSD on echocardiogram, while undergoing (or after recent completion) of ICI therapy, further appropriate work-up (ECG, troponin, BNP or NT-proBNP, C-reactive protein, viral titre, echocardiogram with GLS, cardiac MRI) for ICI-associated CV toxicity, particularly myocarditis and other common differential diagnoses should be carried out promptly [IV, C].
Diretriz Brasileira de Cardio-oncologia – 2020 [14]	Sociedade Brasileira de Cardiologia (SBC)	Brazil (Upper Middle Income)	2020	Para pacientes que desenvolvem novos sintomas cardiovasculares durante ou logo após o tratamento com os ICIs ou que apresentam arritmia, anormalidade do sistema de condução ou disfunção ventricular ao ecocardiograma, recomenda-se iniciar investigação cardiovascular com dosagem de biomarcadores (troponina, NT-proBNP e proteína C reativa), ECG, painel viral, ecocardiograma com strain e RMC para confirmação diagnóstica e exclusão de miocardite viral [IIa, C]. Translation: For patients who develop new cardiovascular symptoms during or soon after treatment with ICIs or who have arrhythmia, conduction abnormality on ECG, or ventricular dysfunction on echocardiogram, it is recommended to initiate cardiovascular investigation with biomarker measurement (troponin, NT-proBNP, and C-reactive protein), ECG, viral panel, echocardiogram with strain, and CMR for diagnostic confirmation and exclusion of viral myocarditis [IIa, C].
Society for Immunotherapy of Cancer (SITC) clinical practice guideline on immune checkpoint inhibitor-related adverse events [15]	Society for Immunotherapy of Cancer (SITC)	USA (High Income)	2021	Patients with suspected ICI-induced myocarditis should undergo cardiac MRI if available (with or without right heart catheterization and myocardial biopsy), EKG, and testing for serum troponin levels.
Management of Immune-Related Adverse Events in Patients Treated With Immune Checkpoint Inhibitor Therapy: ASCO Guideline Update [16]	American Society of Clinical Oncology (ASCO)	USA (High Income)	2021	7.1 Myasthenia Gravis Workup and evaluation: • Consider MRI brain and/or spine depending on symptoms to rule out CNS involvement by disease or alternate diagnosis • Troponin, ECG, and consider TTE and/or cardiac MRI to evaluate concomitant myocarditis (see CV section for further details) 9.1. Myocarditis, Pericarditis, Arrhythmias, Impaired Ventricular Function With Heart Failure, and Vasculitis Workup and evaluation: Additional testing to be guided by cardiology and may include: • Stress test • Cardiac catheterization • Cardiac MRI
Management of Immunotherapy-Related Toxicities, Version 1.2022, NCCN Clinical Practice Guidelines in Oncology [17]	National Comprehensive Cancer Network (NCCN)	USA (High Income)	2022	-
Management of toxicities from immunotherapy: ESMO Clinical Practice Guideline for diagnosis, treatment and follow-up [18]	European Society for Medical Oncology (ESMO)	Europe (High Income)	2022	A diagnostic CMR with inflammatory sequences (T2STIR, T1, LGE) and cardiac troponin are recommended in cases of suspected IR-myocarditis or pericarditis [IV, A].
2022 ESC Guidelines on cardio-oncology developed in collaboration with the European Hematology Association (EHA), the European Society for Therapeutic Radiology and Oncology (ESTRO) and the International Cardio-Oncology Society (IC-OS) [19]	European Society of Cardiology (ESC)	Europe (High Income)	2022	cTn, ECG, and CV imaging (echocardiography and CMR) are recommended to diagnose ICI-associated myocarditis [I, B].

*Abbreviations*: *BNP* Brain natriuretic Peptide, *CMR *cardiac MRI, *cTn* cardiac troponin, *CV *Cardiovascular, *ECG* Electrocardiogram, *GLS* Global longitudinal strain, *ICI* Immune checkpoint inhibitor; *LVSD * Left-ventricular systolic dysfunction, *PET* positron emission tomography, *TTE* Transthoracic echocardiogram [10–16]

As evidenced from multiple national and international clinical practice guidelines, CMR plays a pivotal role in the ICIRM diagnostic landscape. The 2021 Society for Immunotherapy of Cancer (SITC), 2022 European Society of Medical Oncology (ESMO) and 2022 European Society of Cardiology (ESC) guidelines strongly emphasise CMR as diagnostic criterion in suspected ICIRM [12, 15, 16]. However, the 2020 ESMO, 2020 Sociedade Brasileira de Cardiologia (SBC), 2021 American Society of Clinical Oncology (ASCO) guidelines recommend CMR as a confirmatory or additional workup tool, placing less emphasis as compared to the aforesaid [10, 11, 13]. Meanwhile, the 2022 National Comprehensive Cancer Network (NCCN) places no emphasis on the role of CMR [14].

### Barriers to CMR in LMICs

In HICs approximately 26 general MRI scanners are available per million people compared to 1 per million in LMICs. In fact, 66% of LMICs lack access to any MRI imaging [17, 18]. For a Cardiac MRI, scanner cost alone is $1 million, excluding maintenance or consumables like contrast [19]. The lack of inter-sector coordination i.e., public and private as well as specialty coordination i.e., radiology and cardiology in LMICs impact availability of CMR resulting in selective/non-universal affordability [20].

The lack of radiology training posts in many sub–Saharan African and Latin American regions highlight the LMIC deficits [19], with approximately 1.9 radiologists per million inhabitants in LMICs compared to 97.9 in HICs [21]. Africa, as a continent, has fewer MRI machines compared to the USA, by a factor of 30, with almost 40% of these scanners being < 1.0-T systems [22]. The SWiM pilot programme further identified that just under 50% of learners’ facilities in LMICs did not perform CMR despite having the correct hardware, further confirming the lack of penetration of CMR and CMR programmes in LMICs [22].

## Solutions – to increase the role of CMR in LMICs

To increase the role of CMR in LMICs, a comprehensive multipronged approach is imperative. This strategy should involve strong governmental support with policy changes that prioritise healthcare infrastructure, targeting educational initiatives to develop local expertise, and application of novel technology adaptive to its current setting. Addressing these factors will help overcome existing barriers, paving a way for increased penetration of CMR in LMICs [23].

## Governmental support and policy change

Governmental support with policy change can facilitate nation-wide advancements in access to CMR by implementing strict resource allocation and training programmes. Governmental involvement can assist in investments necessary for infrastructure development, such as increasing the number of MRI scanners in Africa, whilst refurbishing current machines. Policy initiatives must promote training and education programmes, like CMR training, embedding these services into national implementation strategies. Prioritisation of CMR with associated funding canincentivise local capacity building, and aid the establishment of cost-effective rapid CMR protocols, adaptive to an LMIC setting [19].

## Education

In an attempt to bridge the gap, the Scan With Me (SWiM) program involves trained professionals from HICs travelling to LMICs and providing educational courses for healthcare professionals on using CMR. A ‘train the trainer’ collaborative structure to improve scan interpretation and utilisation in this case specifically CMR yields rapid training to deliver quality care in LMICs [22].

An example of a cost saving approach is the rapid CMR project, which implemented simplified, less expensive protocols embedded in a training and education programme, spanning 11 LMICs. Scan times for cardiomyopathy were reduced to < 22 min for contrast and < 12 min for non-contrast CMR with 98% images meeting diagnostic quality. Cost reduction was also noted in the range of 30–60%. Mapping this innovation onto the ICIRM scenario would be a significant enabler [19].

## Application of new technology

Finally, the use of artificial intelligence (AI) may reduce scan time along with eliminating need for gadolinium contrast by a method called virtual native enhancement (VNE). This can produce virtual LGE images without the need for contrast. These methods may help keep costs involved with CMR diagnostics to a minimum and may help where radiologists trained in reading CMR scans are not easily available. While these technologies are novel and show good correlation with human-based analysis, more work is needed to prove translation of AI into CMR interpretation [24]. The creation of support networks through the implementation of AI can close the gap, enabling LMICs to receive appropriate diagnostic support from HICs and improve the interpretation of studies in real time.

## Conclusion

The global annual cancer incidence is estimated to be 35 million in 2050, which is a postulated 77% increase from 2022 [25]. Early onset cancers i.e., in people aged < = 50 years, have increased from 1.8 million in 1990 to 3.2 million in 2019 with a projected increase of a further 31% by 2030 [26]. With an ever-rising global cancer burden, novel and/or more indications for ICI therapy, we can expect to see more ICIRM cases along with translation of dynamics from HICs to LMICs. Implementing the gold standard CMR for diagnosis is paramount [27]. Improvising on the innovation of cost-effective, time efficient CMR technology is an access enabler in the LMIC setting – an unmet need, now more than ever.

## Data Availability

No datasets were generated or analysed during the current study.
